# Bilateral Hydronephrosis Secondary to Giant Cell Tumor of the Sacrum

**DOI:** 10.7759/cureus.12099

**Published:** 2020-12-15

**Authors:** Omer Farooq Rehman, Amer K Hussain, Mohammad Ummair, Musab Umair, Muhammad Waqar

**Affiliations:** 1 Urology, Armed Forces Institute of Urology, Rawalpindi, PAK; 2 Surgery, Our Lady of Lourdes Hospital, Drogheda, Cavan, IRL; 3 Orthopedics, North Cumbria University Hospitals NHS Trust, Carlisle, GBR; 4 Urology, California Institute of Behavioral Neurosciences & Psychology, Fairfield, USA; 5 Urology, Kings College Hospital, London, GBR

**Keywords:** giant cell tumor, sacrum, hydronephrosis, bilateral ureteric obstruction, hydronephroureter

## Abstract

Sacral giant cell tumors (GCTs) are rare entities that exhibit slow progressive growth and become clinically apparent when they reach a considerable size. The current case report discusses the presentation, investigation, and management of a young male patient diagnosed with a large sacral mass. A 17-year-old male patient presented with uremia, bilateral lumbar pain, and severe weakness of his lower extremities. Imaging revealed a midline sacral mass causing bilateral upper tract obstruction. The patient underwent bilateral nephrostomies followed by a partial en bloc sacrectomy and curettage of the tumor bulk. Histopathology revealed a giant cell tumor of the sacrum. Postoperatively, the patient received adjuvant radiotherapy and rehabilitation for his neurological symptoms. Sacral GCTs are essentially benign but behave like a malignant tumor in view of frequent recurrences and reports of malignant transformation. Surgery with wide local excision remains the ideal modality for complete clearance of sacral tumors. Nevertheless, limitations include their large size, difficult operative access, risk of fatal intraoperative bleeding, and inevitable high postoperative morbidity.

## Introduction

Giant cell tumors (GCTs) of the sacrum are rare entities. This pathology mainly affects the long bones. In general, the incidence of giant cell tumors is reported at 1%-5% of all bone tumors and is mainly found in the long bones [1-4]. The incidence of sacral GCTs has been reported at 1.7%-8.2% and most commonly in the second to fourth decades of life [5-8]. In order of frequency, the sacrum is the fourth most common site for its occurrence. Metastasis to distant sites is fairly uncommon but pulmonary metastasis has been reported in 3% of cases [1]. It is essentially benign but behaves like a malignant tumor in view of frequent recurrences and reports of malignant transformation.

Sacral GCTs exhibit a silent progression of growth and become clinically apparent after they have reached a considerable size. Wide local resection in these cases imminently results in severely unacceptable neurological deficits. Despite management algorithms having been proposed, the ideal treatment for such tumors needs further deliberation.

## Case presentation

A 17-year-old boy presented to the neurosurgical department with new-onset urinary incontinence and long-standing bilateral lumbar pain along with weakness in his lower extremities. His past history revealed he had difficulty in walking, recurrent urinary tract infections, and worsening constipation for the past two years. On physical exam, he was wheelchair-bound, appeared pale and distressed. On abdominal examination, a non-pulsatile lower abdominal swelling was palpable, approximately 6*8 cm in size, which was non-tender to touch and had a firm to hard consistency. Furthermore, he had bilateral loin and sacral tenderness. On digital rectal exam, the rectum appeared to be displaced by a large swelling compressing the posterior wall of the rectum along with loss of anal tone and diminished perineal sensations. On neurological examination, there was a severe loss of power in both lower extremities (2/5) with bilateral absent ankle reflexes. His baseline investigations revealed abnormalities: hemoglobin of 9.0 mg/dl, serum creatinine levels of 5.4, and urea of 156 mmol/dl. Other lab parameters were within normal limits. Subsequently, an abdominal ultrasound revealed bilateral hydronephroureters and an ill-defined mass extending from the posterior surface of the bladder to the sacrum.

**Figure 1:**
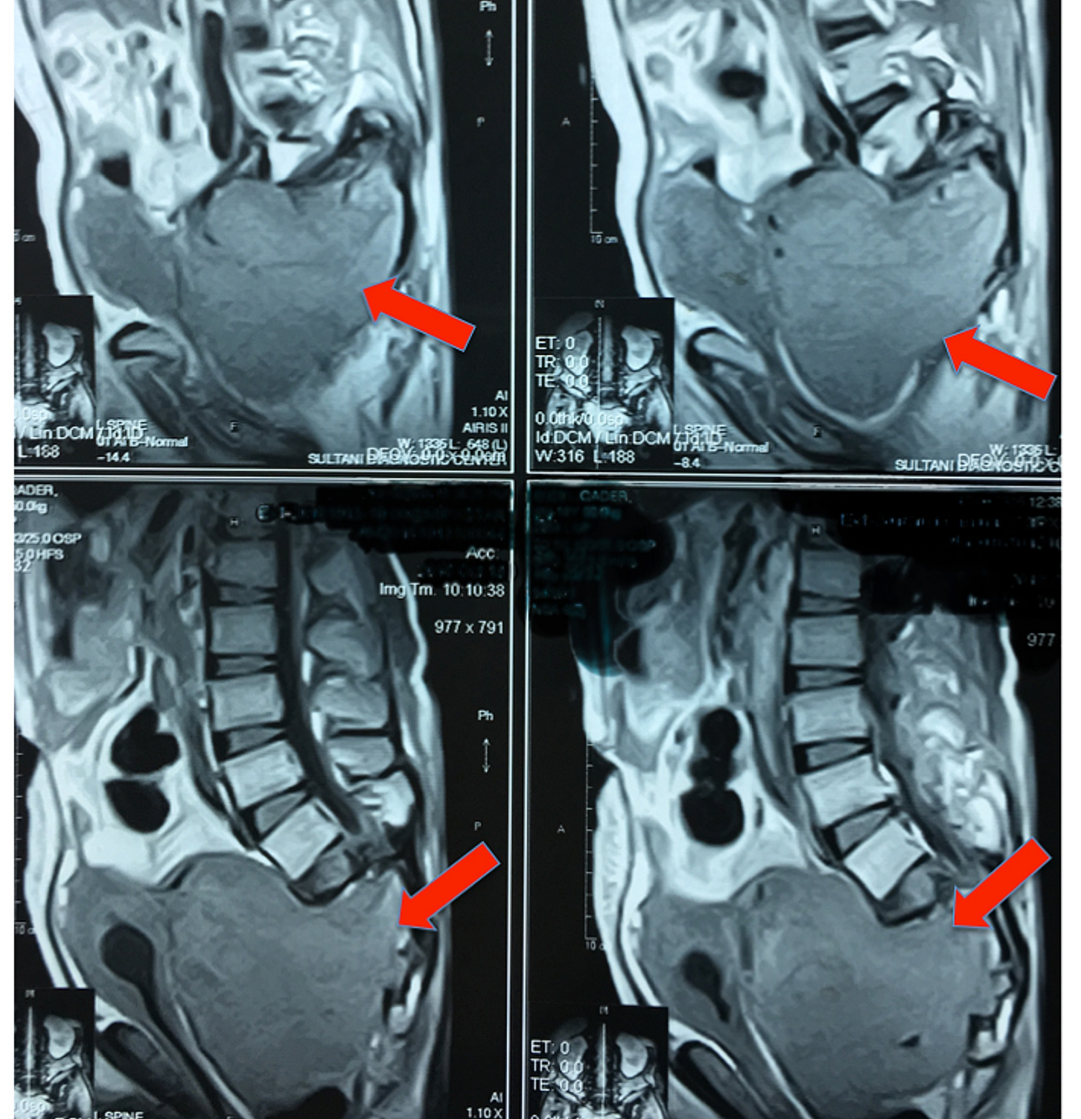
Sagittal MRI image (T1 weighted) of a sacral mass (10.7*10.0*8.3 cm) with near-total destruction of the sacrum, exhibiting local extension The sacral mass projects anteriorly causing forward compression onto the bladder. GCT: Giant Cell Tumor, MRI: Magnetic Resonance Imaging

**Figure 2:**
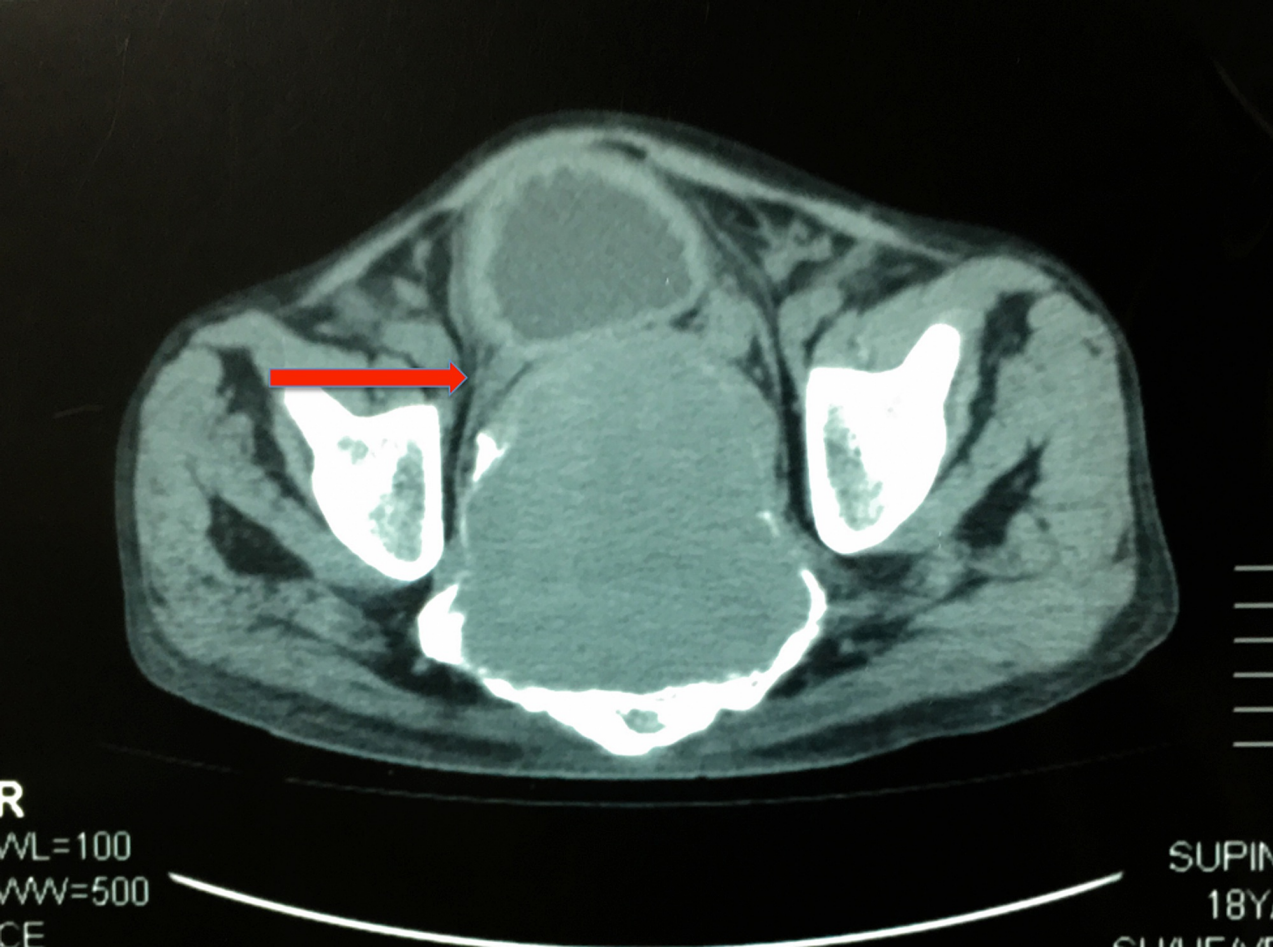
Axial CT image of a large expansile lytic lesion involving the midline sacrum compressing the bladder and ureters anteriorly CT: Computed Tomography

A lumbosacral magnetic resonance imaging (MRI) scan (T1 -weighted) showed a large (10.7*10.0*8.3 cm) expansile lytic lesion, involving the midline sacrum with extensive soft tissue component and ill-defined margins, as shown in Figure 1. The bladder has been pushed anteriorly with compression of the ureters bilaterally, as shown in Figure 2. Bilateral hydronephroureters along with marked parenchymal thinning of both kidneys were documented.

Upon urological consultation bilateral nephrostomies were placed as a urinary diversion. Operatively, a highly vascular, friable, vacuolar mass arising from the sacrum was noted. Partial en bloc sacrectomy and subtotal resection of the tumor was performed due to per operative bleeding. The patient was transfused with four red cell units and two fresh frozen plasma units intraoperatively. The patient was referred to the regional oncology unit for radiotherapy and rehabilitation.

The specimen histopathology report showed the following findings:

Gross specimen: Multiple tan brown hemorrhagic tissue pieces

Microscopy: A tumor composed of sheets of the spindle to mononuclear cells with dispersed. multinucleated osteoclast-like giant cells. No atypia was seen. Rare mitotic activity observed.

Immunochemical stains: CD68 (highlight giant cells), p53 (positive in giant cells).

The long-term risk of recurrence and response to radiotherapy is yet to be established for our patient.

## Discussion

Malignant and primary tumors of the sacrum are rare. Metastasis from distant sites, lymphoma, or multiple myeloma are more common as compared to primary sacral tumors. The differential diagnosis of sacral lesions identified on radiological imaging includes benign conditions such as giant cell tumor, aneurismal bone cyst, osteoid osteoma, osteoblastoma, and nerve sheath tumors. Malignant differentials may include sacral chordoma, chondrosarcoma, Ewing's sarcoma, osteosarcoma, and Paget's sarcoma.

Classically, on CT and MR imaging, GCTs are known to be heterogeneous with areas of low attenuation that represent necrotic foci. Bleeding in the mass is demonstrated by a high signal intensity on T1-T2 weighting imaging. A low-intensity signal on T2 weighting represents areas of dense fibrosis and high hemorrhagic content within the neoplasm. Multiple fluid levels on imaging that represent hemorrhage and sedimentation characterize an aneurismal bony cyst. Osteoid osteomas are rare, with an incidence of 2% in the sacrum. They generally exhibit a low-intensity signal on T1 weighting and an intermediate-level signal on T2. A characteristic finding of osteoblastoma is usually a flare that is representative of an inflammatory response around the tumor, however, it is generally considered to have a nonspecific representation on MR. Hemangiomas of the sacrum depict a classical honeycomb appearance on imaging, as well as coarse trabecular thickening.

Essentially, the management of sacral GCTs is surgical, non-surgical, or a combination of both. Surgical approaches include intra-lesional curettage, sclerotherapy, and subtotal or total en-bloc sacrectomy, whereas non-surgical options enlist selective arterial embolization (SAE), cryotherapy, radiotherapy, and, more recently, monoclonal antibodies.

Selective arterial embolization of the sacral tumor-feeding arteries has shown promising results in terms of limiting the tumor progression of sacral GCTs. Hosalkar et al. published a series of nine patients of which seven showed no progression at a mean follow-up of 8.96 years [9]. The limitations of SAE include the risk of diagnostic error, as only a needle biopsy can be used for preoperative diagnosis. Due to the lack of expertise and need for repeated treatment, SAE was not a feasible treatment option in our current setup.

Radiotherapy and cryotherapy are other non-surgical modalities used for the treatment of sacral GCTs. Due to the high rate of local recurrence, these techniques are not preferable as a sole primary treatment. Leggon et al. [10] reported a 62% rate of recurrence with the use of cryotherapy, whereas recurrence rates with radiotherapy are as high as 49%. Recent advancements include the use of monoclonal antibodies in the preoperative management of large-sized sacral GCTs. Denosumab is a monoclonal antibody that blocks the RANKL-RANK pathway and, as a result, interferes with osteoclast-mediated bone destruction. Specifically, denosumab has shown to decrease CT enhancement of the tumor, as well as, aid in reducing intraoperative blood loss by approximately two liters [11].

The treatment management of GCTs of the spine and sacrum is complex in nature. Surgical excision with wide local margins remains the ideal option for complete removal of the sacral tumor, with reports of a 0% risk of recurrence [10]. Nevertheless, limitations, including their large size, difficult operative access, risk of fatal intraoperative bleeding, and high postoperative morbidity, make surgery a difficult choice [12]. There is a considerable risk of a microscopic residual tumor if the sacral nerves are preserved. Literature reports recurrence rates of up to 50% if an adequate margin size is not taken into account while performing a subtotal or complete en-bloc sacrectomy [13]. Whereas if the sacral nerve roots are compromised, there is a high likelihood of bowel and bladder dysfunction. Furthermore, the challenges of spino-sacral reconstruction have to be taken into account.

## Conclusions

The management of sacral GCTs requires a multidisciplinary approach. Complete resection is the treatment of choice; however, radiotherapy serves as a suitable adjuvant therapy in cases of subtotal resection. Close monitoring is required to assess the risk of local recurrence and metastasis.
